# Integrative analysis of single-cell and bulk multi-omics data to reveal subtype-specific characteristics and therapeutic strategies in clear cell renal cell carcinoma patients

**DOI:** 10.7150/jca.101451

**Published:** 2024-10-18

**Authors:** Xinjia Ruan, Chong Lai, Leqi Li, Bei Wang, Xiaofan Lu, Dandan Zhang, Jingya Fang, Maode Lai, Fangrong Yan

**Affiliations:** 1 State Key Laboratory of Natural Medicines, Research Center of Biostatistics and Computational Pharmacy, China Pharmaceutical University, Nanjing 211198, P.R. China.; 2 Department of Urology, The First Affiliated Hospital, Zhejiang University School of Medicine, Hangzhou 310012, P.R. China.; 3 School of Mathematical Sciences, Jiangsu Second Normal University, Nanjing 210013, P.R. China.; 4 Department of Pathology, Zhejiang University School of Medicine, Hangzhou 310058, P.R. China.

**Keywords:** Clear cell renal cell carcinoma, Multi-omics, Single cell, Immune infiltration, Precision treatments

## Abstract

**Background:** Kidney renal clear cell carcinoma (KIRC) is the most prevalent subtype of malignant renal cell carcinoma and is well known as a common genitourinary cancer. Stratifying tumors based on heterogeneity is essential for better treatment options.

**Methods:** In this study, consensus clusters were constructed based on gene expression, DNA methylation, and gene mutation data, which were combined with multiple clustering algorithms. After identifying two heterogeneous subtypes, we analyzed the molecular characteristics, immunotherapy response, and drug sensitivity differences of each subtype. And we further integrated bulk data and single-cell RNA sequencing (scRNA-Seq) data to infer the immune cell composition and malignant tumor cell proportion of subtype-related cell subpopulations.

**Results:** Among the two identified consensus subtypes (CS1 and CS2), CS1 was enriched in more inflammation-related and oncogenic pathways than CS2. Simultaneously, CS1 showed a worse prognosis and we found more copy number variations and BAP1 mutations in CS1. Although CS1 had a high immune infiltration score, it exhibited high expression of suppressive immune features. Based on the prediction of immunotherapy and drug sensitivity, we inferred that CS1 may respond poorly to immunotherapy and be less sensitive to targeted drugs. The analysis of bulk data integrated with single-cell data further reflected the high expression of inhibitory immune features in CS1 and the high proportion of malignant tumor cells. And CS2 contained a large number of plasmacytoid B cells, presenting an activated immune microenvironment. Finally, the robustness of our subtypes was successfully validated in four external datasets.

**Conclusion:** In summary, we conducted a comprehensive analysis of multi-omics data with 10 clustering algorithms to reveal the molecular characteristics of KIRC patients and validated the relevant conclusions by single-cell analysis and external data. Our findings discovered new KIRC subtypes and may further guide personalized and precision treatments.

## Introduction

Renal cell carcinoma (RCC) is a type of kidney cancer that is highly heterogeneous within or between tumors. From a clinical perspective, there are three major histologic subtypes of RCC: papillary (pRCC), chromophobe (chRCC), and clear cell renal cell carcinoma (ccRCC) 1. ccRCC, also known as kidney renal clear cell carcinoma (KIRC), is the most common subtype, accounting for approximately 80-90% of all RCC cases. It is also the leading cause of death from kidney cancer 2. Surgical resection remains the main treatment modality for most patients with KIRC. However, metastatic recurrence occurs in 30-40% of patients with localized lesions during their follow-up after surgical resection 3. Based on clinicopathological features, KIRC is classified into four grades according to the Fuhrman scoring system and four stages according to the American Joint Committee on Cancer staging, which are associated with prognosis 4, 5. The identification of molecular subtypes of KIRC may facilitate the implementation of precision medicine and be a potential prognostic indicator in KIRC patients.

Genomic and molecular studies of KIRC have greatly aided diagnosis and treatment. Recurrent and advanced KIRC patients may exhibit insensitivity to traditional chemoradiotherapy 6. Vascular endothelial growth factor (VEGF) is an effective drug target for patients with metastatic kidney cancer, and multitargeted tyrosine kinase inhibitors (TKIs) that inhibit VEGFR are key treatments for patients with advanced or metastatic KIRC 7. Some advanced KIRC patients also could benefit from immune checkpoint inhibitors (ICIs), but most patients still experience spontaneous or acquired treatment resistance. The mechanisms of ICI resistance are influenced by tumor cells and their microenvironment 8. These indicated that the response of KIRC patients to targeted therapy or immunotherapy varies among individuals. Therefore, identifying subtypes of KIRC patients may help to unravel the mechanisms of cancer progression and choose a personal therapeutic strategy for KIRC patients.

In a previous study, Hu *et al.* 9 and Chen *et al.* 10 classified KIRC patients into multiple subtypes based on a multi-omics dataset, and customized disease treatment strategies according to the differences among these subtypes. In addition, there have been several studies of KIRC typing based on signature genes to explore subtypes with specific molecular biomarkers, such as immune- and inflammation-associated genes and ferroptosis-related lncRNAs 11-13. However, no KIRC typing study has thus far explored the combination of multi-omics with multiple clustering algorithms, and integrated single cell data to support the analysis.

Integrating multi-omics information can provide more relevant evidence for biological mechanisms, thereby revealing complex biological processes. A variety of clustering algorithms can help derive more stable and robust subtypes. On the other hand, single-cell sequencing technology has developed rapidly in recent years and can characterize gene expression and genomic information at the level of individual cells 14. It has been shown to have obvious advantages in identifying cellular heterogeneity. Combining single-cell data with bulk data can identify phenotype-related cell subpopulations 15, which aids in a deeper understanding of the unique molecular characteristics exhibited by KIRC subtypes.

In this study, we conducted a comprehensive multi-omics analysis of the genomics, transcriptomics, and epigenomics of KIRC using 10 clustering algorithms to reveal the subtype-specific molecular characteristics of KIRC patients. We used the subtype information obtained from bulk data to identify cell subpopulations that were highly associated with them, and revealed the differences between the two at the single-cell level. Then, we verified the reliability of the classification with external datasets. Furthermore, we discussed potential clinical treatment strategies based on specific molecular features, including targeted therapy and ICI drugs.

## Materials and Methods

### Patients and samples

Samples had been given by The Cancer Genome Atlas (TCGA). “TCGAbiolinks” 16 R package was used for obtaining transcriptome expressions from TCGA-KIRC cohort. RNA-seq data based on TCGA then preprocessed for eliminating genes with low expressions, preserving those with CPM of ≥ 1 in at least 10% of the samples. The GENCODE 27 file was utilized to annotate mRNAs that had been filtered. The protein-coding genes were maintained, and Vega (https://vega.archive.ensembl.org/) was used to identify lncRNAs. The number of nonoverlapping exons for every thousand bases for every million mapped segments (FPKM) was then computed and translated to transcript values for every million bases (TPM). TPM expressions for lncRNA and mRNA had been transformed using Log2 computations. The methylation data obtained by TCGA was used the Infinium 450K array. Clinical information had been retrieved from Xena Public Data Hubs, and somatic mutation information had been downloaded from Firehose.

Upon matching gene expressions, mutation, methylation, clinical data, and copy number variation data of 538 KIRC individuals, twenty-three samples with molecular pathological features of MiTF/TFE translocation RCC, suspicious RCC, and clear cell papillary RCC were excluded 17. Multi-omics data of 252 individuals had been eventually included for follow-up analysis.

External validation cohorts utilized to construct the mRNA expression matrix and clinical information were MTAB1980, GSE150404, GSE73731, and GSE40435 18-20. Supplementary Table S1 summarizes the sample sizes, platforms, and tissue sources for these cohorts.

The scRNA-seq data came from GSE222703 21, which included tumor and adjacent healthy tissue samples from 3 patients with renal clear cell carcinoma. We utilized quality-controlled and annotated data for subsequent analyses.

### Identification of molecular subtypes

We defined subtypes of KIRC patients according to lncRNA and mRNA expressions, somatic mutation, and DNA methylation through R package "MOVICS" 22. We chose elite features, such as 1,000 lncRNAs, 1,500 mRNAs, and 1,500 DNA CpG methylation sites, as well as mutant genes having mutation rates > 0.30. The appropriate number of subtypes was then determined by examining clustering prediction index (CPI) and gap statistics 23 according to multi-omics. The clustering effect is enhanced with increasing CPI and gap statistics values. Clustering was then performed using ten advanced multi-omics clustering algorithms: SNF, iClusterBayes, PINSPlus, LRAcluster, NEMO, IntNMF, COCA, ConsensusClustering, MoCluster, and CIMLR. Default parameters were used. Combined classifications were produced by a consensus set obtained from function "getConsensusMOIC()",while subtypes had been recognized with considerable reliability. Using silhouette scores, the similarity of subtype samples was assessed.

### Gene expression analysis and pathway enrichment analysis

The R package “DESeq2” 24 was used to detect differentially expressed mRNAs using raw counts from expressions of RNA-seq genes, with the filtering parameters set to false discovery rate (FDR) < 0.05 and |log2 Fold Change (log2 FC)| > 2.

For pathway functional enrichment, we used the "clusterprofiler" R package 25, and *P* values were corrected for multiple testing using the Benjamini-Hochberg method with FDR <0.05. We assessed the levels of activation for interesting pathways, including the published oncogenic pathways 26 and immune cell features 27 to further elucidate the specific characteristics of each subtype. For the pathways of interest, single-sample gene set enrichment scores were calculated using the R package "GSVA" 28, and heatmaps displayed the average enrichment scores of each subtype.

### Characterization of genetic alterations in subtypes

We downloaded somatic copy number alteration (SCNA) data from Firehose and performed SCNA analysis by using GISTIC2.0 on GenePattern 29. Subsequent steps include annotating the reference genome file (hg19.mat) and exploring genomic regions with significant amplifications or deletions. Then the differences in mutation frequencies among individual samples were estimated by using R package “maftools” 30, as well as mutations with significant differences were identified. And an overall mutation landscape map was produced by function "Oncoprint()".

We determined TMB by calculating the number of nonsynonymous mutations for every million bases. FGA denotes the percentage of genome impacted by a copy number change.

### Immune microenvironment analysis

To characterize the tumor microenvironment, we assessed the infiltration of stromal and immune cells and tumor purity from the expression data by using the ESTIMATE algorithm 31. Moreover, we estimated the population abundance of infiltrating immune and stromal cell populations by MCPcounter 27.

We also applied the CIBERSORTx online analysis platform to complete the immune microenvironment analysis 32. We first constructed the single cell expression matrix and built the scRNA-Seq signature matrix according to the instructions with CIBERSORTx. The single-cell reference data were derived from a publicly published ccRCC dataset 33. We screened untreated tumor samples and reclassified cell types. Then we uploaded the mixture datasets of KIRC bulk tissue gene expression profiles and chose the signature matrix we obtained before. We selected “S-mode” for batch correction and set permutations to 100. Other parameters retained the default. Finally, we obtained the relative proportions of 16 subsets of tumor-infiltrating immune cells in each sample.

Then, we uploaded the KIRC combination of bulk tissue gene expression profile datasets and chose the previously produced signature matrix. We selected "S-mode" for batch correction with 100 permutations. The remaining parameters were left at their default values. Finally, the relative proportions of sixteen subgroups of tumor-infiltrating immune cells in each sample were identified.

### Assessment of the response to immunotherapy

We used the tumor immune dysfunction and exclusion (TIDE) algorithm 34 to predict the likelihood that an individual will respond to immunotherapy. A higher TIDE score suggests increased dysfunction and T cell rejection by immunological microenvironment, indicating a decreased chance of benefiting from immune checkpoint blockade (ICB). We evaluated the immunotherapy response status for each sample by using the TIDE online application.

### Integration analysis of bulk and scRNA-Seq data

We did data preprocessing and performed scRNA-seq data analysis by Seurat V4.2.0 35. We first removed low-quality cells and filtered out lowly expressed genes. Subsequently, we normalized the expression matrix using the "NormalizeData()" function with default parameters. Highly variable genes were identified using the "FindVariableFeatures()" function with the "vst" method. We then performed data scaling with the "ScaleData()" function, and conducted principal component analysis (PCA) and UMAP dimensionality reduction. We used SCISSOR 36 to associate gene expression and phenotypic data from TCGA with the single-cell RNA-seq data of clear cell renal cell carcinoma samples. SCISSOR was run on each patient individually according to the scissor tutorial for logistic regression with the alpha-parameter of 0.2. Scissor+ and Scissor- cells represented CS1-like cells (Scissor_CS1) and CS2-like cells (Scissor_CS2), respectively.

### Inference of copy number variations from scRNA-seq data

InferCNV (https://github.com/broadinstitute/inferCNV/wiki) is an effective analytical tool for inferring malignant tumor cells in single cells, and it was used to explore copy number alterations of single cell RNA-Seq data. We used immune cells as the set of reference “normal” cells and run inferCNV analysis by the following parameters: "denoise" was TRUE, "HMM" was TRUE, "HMM_type" was set "i6", and a value of 0.1 for "cutoff".

### Chemotherapeutic response prediction

Using the "pRRophetic" R package and Genomics of Drug Sensitivity in Cancer (GDSC) database 37, we made predictions on therapeutic responses per sample to targeted drugs. "AllSoldTumours" tissue type was selected for study, and the ComBat function was used to remove cell lines' batch effects. For repeated gene expression measurements, the average value was used, and all other parameters were kept at their defaults. We estimated half maximal inhibitory concentration (IC_50_) of samples by using ridge regression for fitting the homogenized dataset, with a higher IC_50_ suggesting less drug sensitivity. And the prediction accuracy was assessed through 10-fold cross-validation on the training set for GDSC, producing estimates of sensitivity for every chemotherapeutic drug.

### Validation of external cohorts

To predict the robustness of subtypes in validation cohorts, we used "nearest template prediction" (NTP) 38, which may capture the direction of expression change of signature genes in the modeling of templates without optimization of the analysis parameters. NTP first defines the template of the subtype as a representative expression pattern of the characteristic genes of each subtype based on the predetermined gene signature of each subtype, which are the n overexpressed genes in the subtype. Then, from the microarray data of the measured genes in the samples, the signature genes were extracted, and their proximity to the templates was evaluated by calculating the distance. Finally, the label of the closer template is assigned as a predicted result of the sample.

### Statistical analyses

Our statistical analyses were performed based on R (v.4.1.1). We used fisher's exact test of independence or chi-squared to statistically test the correlation between categorical clinical data and defined subtypes. For continuous data, we used the Mann-Whitney test. We performed survival analysis using the R package “survival” and assessed differences in overall survival (OS) between subtypes using Kaplan-Meier plots and log-rank tests. For all statistical analyses, P values less than 0.05 were considered as having significance statistically.

## Results

### Two subtypes of KIRC established by the multi-omics classification

**Figure 1:**
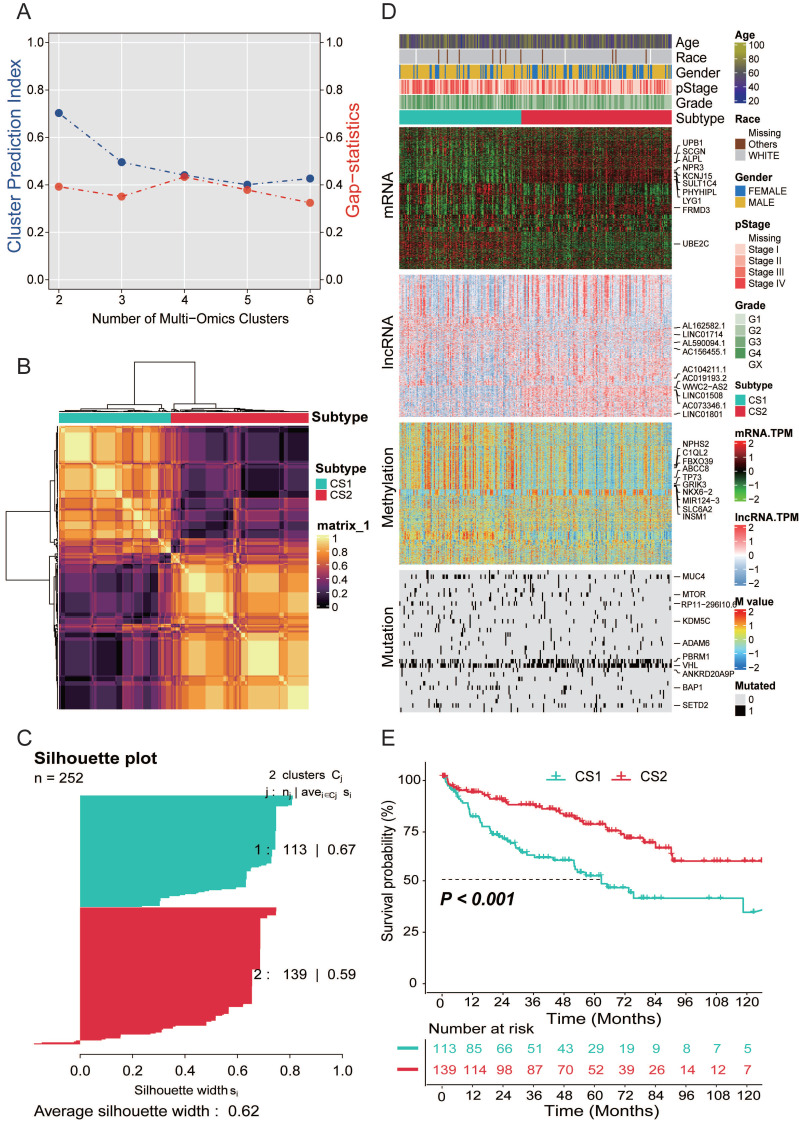
**(A)** The optimal number of clusters was determined by calculating the CPI (blue line) and gap statistics (red line) of the KIRC cohort. **(B)** Consensus heatmap based on the results of 10 multi-omics advanced clustering algorithms with a cluster number of 2. **(C)** The silhouette plot shows sample similarity by using silhouette scores based on the consensus clustering results. **(D)** Comprehensive heatmap of multi-omics integrative clustering by 10 clustering algorithms with annotation of the top features.** (E)** Kaplan‒Meier survival analysis of overall survival in the two subtypes.

After matching multi-omics data, 252 samples were included in the follow-up analysis. To find the optimal number of the subtypes, we assessed the gap statistics and CPI based on multi-omics data. The CPI peaked at k=2, thus we selected 2 as the final number of clusters (Fig. 1A). Subsequently, in order to make the classification results more reliable, we clustered through 10 advanced multi-omics clustering algorithms, finally obtained subtype grouping through consensus clustering, and divided the patients into 2 subtypes: CS1 and CS2 (Fig. 1B). We also quantified sample similarity within subtypes by silhouette scores. The results showed good separation and differentiation between the two subtypes, with contour scores of 0.67 and 0.59 for each subtype, respectively (Fig. 1C).

As shown in Fig. 1D, we provided the distribution of multi-omics data for each subtype. Heatmaps of mRNAs reveal that every isoform can be clearly distinguished. In the representative omics datasets, we presented the elite features with a high impact on subtyping. In addition, information on patient subtype, age, gender, race, pathological stage and grade was also listed.

### Survival analysis and clinical characteristics

The results of the survival analysis showed a significant difference in overall survival between the two subtypes (*P*<0.001; Fig. 1E). The prognosis of CS2 was better, and the OS of CS1 was significantly lower than that of CS2.

We compared the clinical variables of patients with the two subtypes, including gender, ethnicity, pathological stage, grade, and age in Table 1. We found that significant differences in patient gender, with more female patients present in the CS2 subtype (*P*=0.001). The pathological staging results showed that more CS2 patients had stage I and II disease (*P*=0.006). The grade results showed that CS1 patients were more likely to be in higher grades (*P*<0.001).

### Differentially expressed genes found by pathway enrichment analysis

We performed differential expression analysis by DESeq2 and identified 1,139 significantly differentially expressed genes with a threshold FDR less than 0.05 and an absolute value of log2FC greater than 2 (Supplementary Table S2). In CS2, which has a better prognosis, 88 genes were significantly upregulated, and 1,051 genes were significantly upregulated in the CS1 subtype. Among these genes, we found that 8 genes (CTXN3, SST, CYP1D1P, SLC6A19, TMEM174, SLC6A18, SCGN, and RGS7) were upregulated more than 3-fold in CS2, and 14 genes (HP, PAEP, SAA2-SAA4, FDCSP, AL391095.2, SAA1, LBP, IGFBP1, SAA2, AC091812.1, KCNS1, HPR, ANGPTL8 and AL161431.1) were upregulated more than 5-fold in CS1.

Next, we performed pathway enrichment analysis of differentially expressed genes in the CS1 with worse prognosis. The results of GSEA pathway enrichment analysis of hallmark gene sets showed that epithelial mesenchymal transition and hypoxia was enriched in the CS1 subtype, a variety of signature inflammatory features, such as inflammatory response and IL6/JAK/STAT3 signaling pathways were upregulated in CS1 (Supplementary Fig. 1A). In the results of KEGG enrichment analysis, we also found that the chemokine signaling pathway, IL-17 signaling pathway, and other inflammation-related pathways were upregulated in CS1, while the TGF-beta and Wnt signaling pathways were also enriched in CS1 (Supplementary Fig. 1B).

### Genetic alteration of the two subtypes

**Figure 2:**
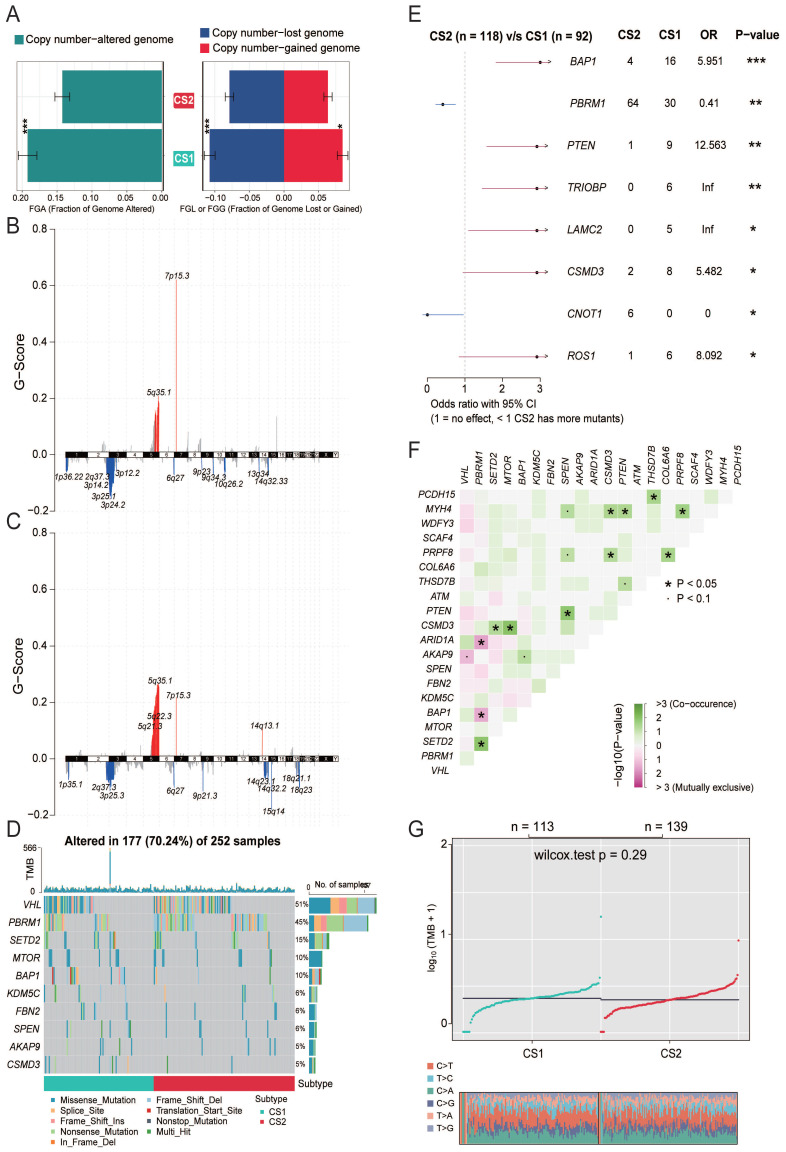
** (A)** Bar plot of fraction genome altered between the two subtypes. **(B)** Copy number amplifications and deletions of the 23 chromosomes in CS1. **(C)** Copy number amplifications and deletions of the 23 chromosomes in CS2. **(D)** Waterfall plot shows the somatic mutation landscape of the top 10 most frequently mutated genes. The bars above the heatmap represent the number of mutations occurring for each subject, and the bars on the right show the number of subjects having a mutation for each gene. **(E)** The forest plot displays the significantly differentially mutated genes between the two subgroups. **(F)** The heatmap shows the mutually cooccurring and exclusive mutations of the top 20 frequently mutated genes. **(G)** Comparison of TMB and TiTv (transitions and transversions) between the two subtypes.

Copy number alterations and gene mutations play a key role in tumorigenesis and development of cancer. Therefore, we need to explore the differences in genetic alterations of patients with different subtypes. We assessed copy number changes and found that CS2 had fewer CNAs than CS1 isoforms in both the lost and gained genomes (Fig. 2A). Then, we analyzed changes in their chromosomal regions by using GISTIC 2.0 and plotted copy number amplifications and deletions based on G-score (Fig. 2B, 2C). We found 3p deletions in the CS1 and CS2, which is associated with the most commonly mutated genes in KIRC (VHL, PBRM1, SETD2, and BAP1). At the same time, it also showed that 5q amplification and 14q deletion were common in KIRC. However, C1 has more significant 7p amplification than CS2, and CS1 has 9q, 10q, 13q deletions, while CS2 has 15q, 18q deletions, and 14q amplifications.

Gene function is affected not only by expression level and copy number variation but also by gene mutation. As shown in Fig. 2D, somatic mutation analysis revealed that 177 (70.24%) of 252 patients with KIRC had mutations, most of which were missense mutations. The overall mutation rate of the 10 mutated genes was > 5%. VHL, PBRM1, SETD2, and BAP1 were the top five mutated genes in KIRC samples, with VHL mutation being the most common mutation. Mutation comparison of the two subtypes showed that there were more BAP1 mutations in CS1 and significantly more PBRM1 mutations in CS2 (Fig. 2E). Previous studies have shown that both BAP1 and PBRM1 mutations are associated with worse outcomes in KIRC patients, but patients with BAP1 mutations have poorer prognosis, higher grades, and lower overall survival than patients with PBRM1 mutations or with no BAP1 mutations. This is consistent with our conclusion that the CS1 subtype has a worse prognosis. We analyzed the mutual exclusion and cooccurrence relationships of mutations. The results showed that most mutations occurred simultaneously, whereas PBRM1 and BAP1 were mutually exclusive (Fig. 2F). Supporting a negative correlation between the presence of BAP1 and PBRM1 mutants, co-mutation of PBRM1 and BAP1 is rare.

In recent years, tumor mutational burden (TMB) has emerged as a promising and clinically validated biomarker for immune checkpoint inhibitors. However, in our study, no significant difference in TMB between the two subtypes was observed (*P*=0.29, Fig. 2G).

### Correlation between subtypes and immune infiltration

**Figure 3:**
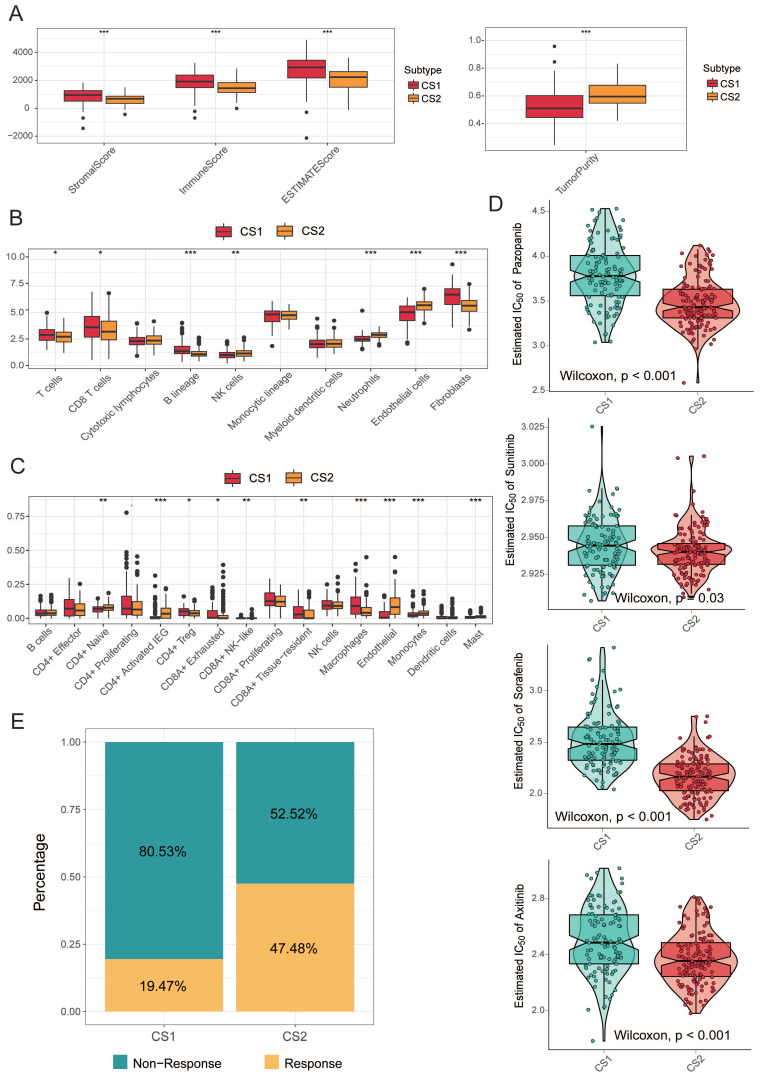
** (A)** The boxplot shows the ESTIMATE evaluation results of different subtypes.** (B)** The boxplot shows the abundance of 10 immune infiltrating cells calculated by the MCP-counter algorithm in different subtypes. **(C)** The boxplot shows the infiltration of some immune cells evaluated by CIBERSORTx in the two subtypes.** (D)** Box plots of the estimated IC_50_ for common TKI drugs (sorafenib, sunitinib, axitinib and pazopanib) of clear cell renal cell carcinoma between the two subtypes. **(E)** Bar plot of immunotherapy responders and non-responders predicted by the TIDE method.

While tumors with high TMB levels are more likely to be recognized by the immune system and respond to immunotherapy, we cannot ignore the impact of the immune microenvironment. We therefore compared differences in immune cell infiltration. The ESTIMATE evaluation results showed that CS1 had a higher immune score and CS2 had a higher tumor purity (Fig. 3A).

Next, we used MCPcounter to calculate the abundance of 10 immune infiltrating cells to compare differences in immune cell infiltration (Fig. 3B). The results showed that CS1 had a significantly higher immune enrichment score in T cells, CD8 T cells, B cells, and fibroblasts. In contrast, CS2 had higher immune enrichment scores for NK cells, neutrophils, and epithelial cells.

Considering that the above reference immune gene sets are derived from mixed tumor samples, and the immune cell types are not sufficiently classified. Therefore, we used a single-cell RNA-Seq signature matrix of renal clear cell carcinoma to determine cell type abundance in bulk RNA-Seq data of KIRC samples by CIBERSORTx analysis. CIBERSORTx is an extended version of CIBERSORT, which has advantages in inferring cell-type-specific gene expression profiles. The evaluation of estimated cell fractions showed that some suppressive immune signatures, such as regulatory T cells (Tregs), CD8A+ exhausted T cells, and tumor-associated macrophages (TAMs), exhibited higher infiltration in the CS1 subtype (Fig. 3C). This may be a potential reason why CS1 has a higher immune enrichment score in T cells and CD8 T cells but is difficult to respond to immunotherapy in immune prediction.

### Precision treatment recommendations for KIRC patients

We analyzed the drug sensitivity of common targeted drugs for clear cell renal cell carcinoma, including sorafenib, sunitinib, axitinib, and pazopanib. We trained prediction models on the GDSC cell line dataset using ridge regression and assessed the accuracy of predictions by 10-fold cross-validation, based on which we estimated IC50 values for each sample in each subtype. As shown in Fig. 3D, the drug sensitivity analysis results demonstrated that the CS2 subgroups all showed lower IC50 values, indicating that they may be more sensitive to TKI drugs.

We further explored the response of the two subgroups of patients to immunotherapy. We used the TIDE algorithm for prediction and found 22 patients in CS1 and 66 patients in CS2 were potentially responsive to immunotherapy (Fig. 3E; 19.47% and 47.48%, respectively; *P*<0.001).

### Differences in immune cell composition of Scissor cell subpopulations

**Figure 4:**
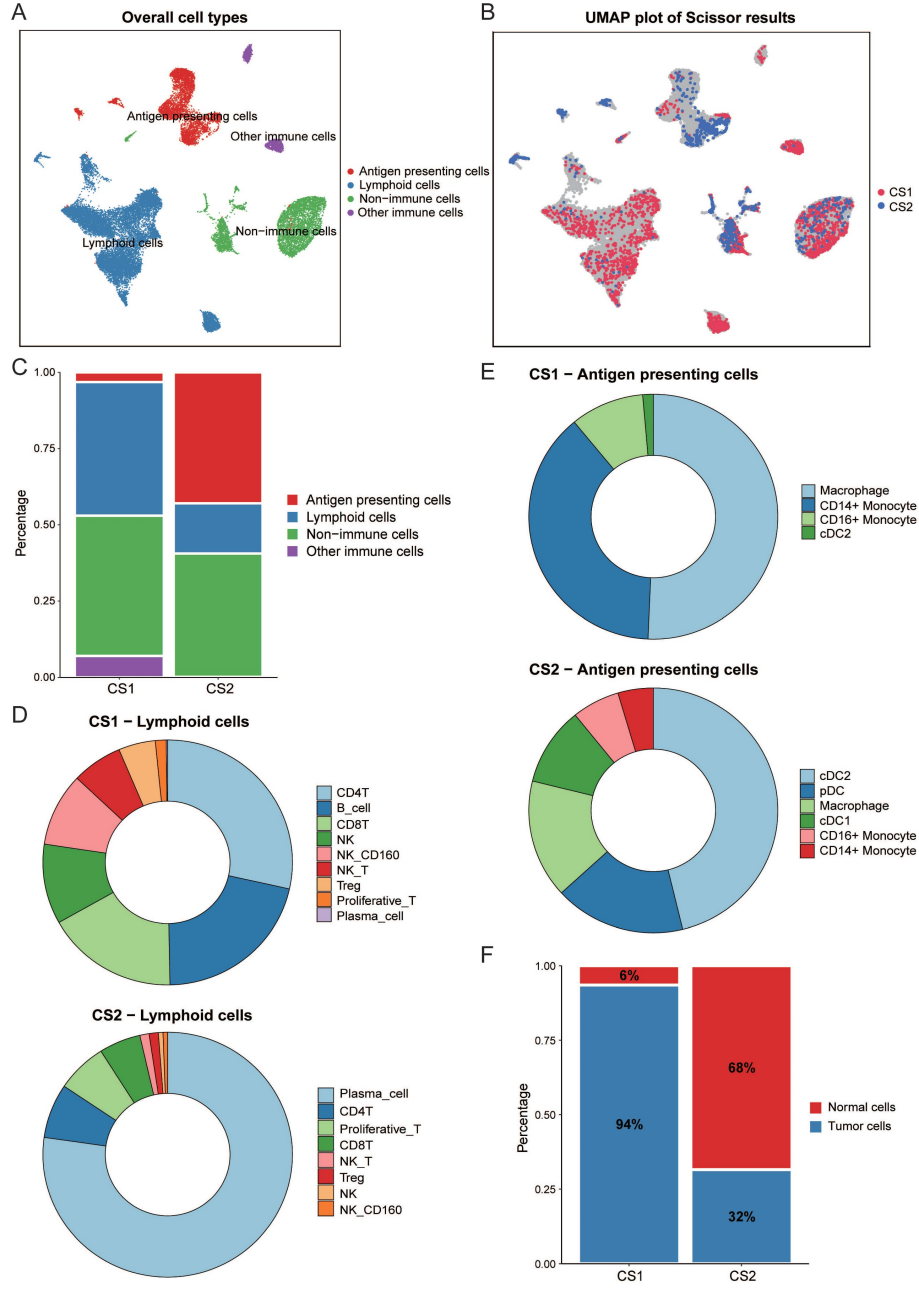
** (A)** UMAP visualization of all cells with annotated clusters. **(B)** UMAP visualization of the selected Scissor cells. The red and blue dots are cells associated with the CS1 and CS2 phenotypes, respectively. **(C)** Main cell composition of Scissor_CS1 and Scissor_CS2 cell subpopulations. **(D)** Distribution of immune-related cell types in Scissor_CS1 and Scissor_CS2.** (E)** The composition of antigen presenting cells in Scissor_CS1 and Scissor_CS2. **(F)** Bar plot of the proportion of malignant cells in Scissor_CS1 and Scissor_CS2.

We performed an integrated analysis of bulk data and single-cell RNA data using Scissor (Fig. 4A, 4B). This is a new method for single-cell data analysis that uses phenotypes of bulk data to identify cell subpopulations most relevant to the phenotype from single-cell sequencing data. A total of 2291 Scissor+ cells (Scissor_CS1) associated with the CS1 subtype and 1013 Scissor- cells (Scissor_CS2) associated with the CS2 subtype were identified. Fig. 4C showed that the Scissor_CS1 cell subpopulation was mainly composed of lymphocytes and non-immune cells, while the Scissor_CS2 cell subpopulation was mainly derived from antigen presenting cells and non-immune cells. We then further studied the detailed distribution of immune-related cell types. We observed that the most abundant lymphocytes in Scissor_CS1 were CD4T, B cells, and CD8T; while in Scissor_CS2, differentiated and mature plasma cells accounted for the largest proportion, followed by CD4T and proliferative T cells (Fig. 4D). Scissor_CS1 and Scissor_CS2 also had great differences in the composition of antigen presenting cells. Scissor_CS1 mainly contained macrophages and monocytes; while in Scissor_CS2, dendritic cells, as the most effective antigen presenting cells (Fig. 4E), accounted for an important proportion and may play a key role in mediating immune responses. Combined with the higher proportion of Treg cells in Scissor_CS1, the Scissor_CS1 cell subpopulation showed potential immunosuppressive characteristics, while Scissor_CS2 showed an activated immune environment. This may be the reason why the CS2 subtype predicted better immunotherapy response results.

### Inference of malignant tumor cells in subtype-related cell subpopulations

For non-immune cells, in order to separate tumor cells from non-malignant cells, we used inferCNV to estimate the copy number variation of individual cell from gene expression data. As shown in supplementary Fig. 2A and 2B, Scissor_CS1 had more copy number variation. Then, a threshold of 0.15 was set based on the copy number alteration (CNA) score in non-immune cells and the observed bimodal distribution (Supplementary Fig. 3A), and normal cells and malignant tumor cells in two cell subpopulations were inferred. It can be seen from Fig. 4F that the proportion of malignant cells in the Scissor_CS1 cell subpopulation is significantly higher than that in Scissor_CS2 (P < 0.001). This is consistent with the worse prognosis of the CS1 subtype.

### Validation of subtypes in four external cohorts

**Figure 5:**
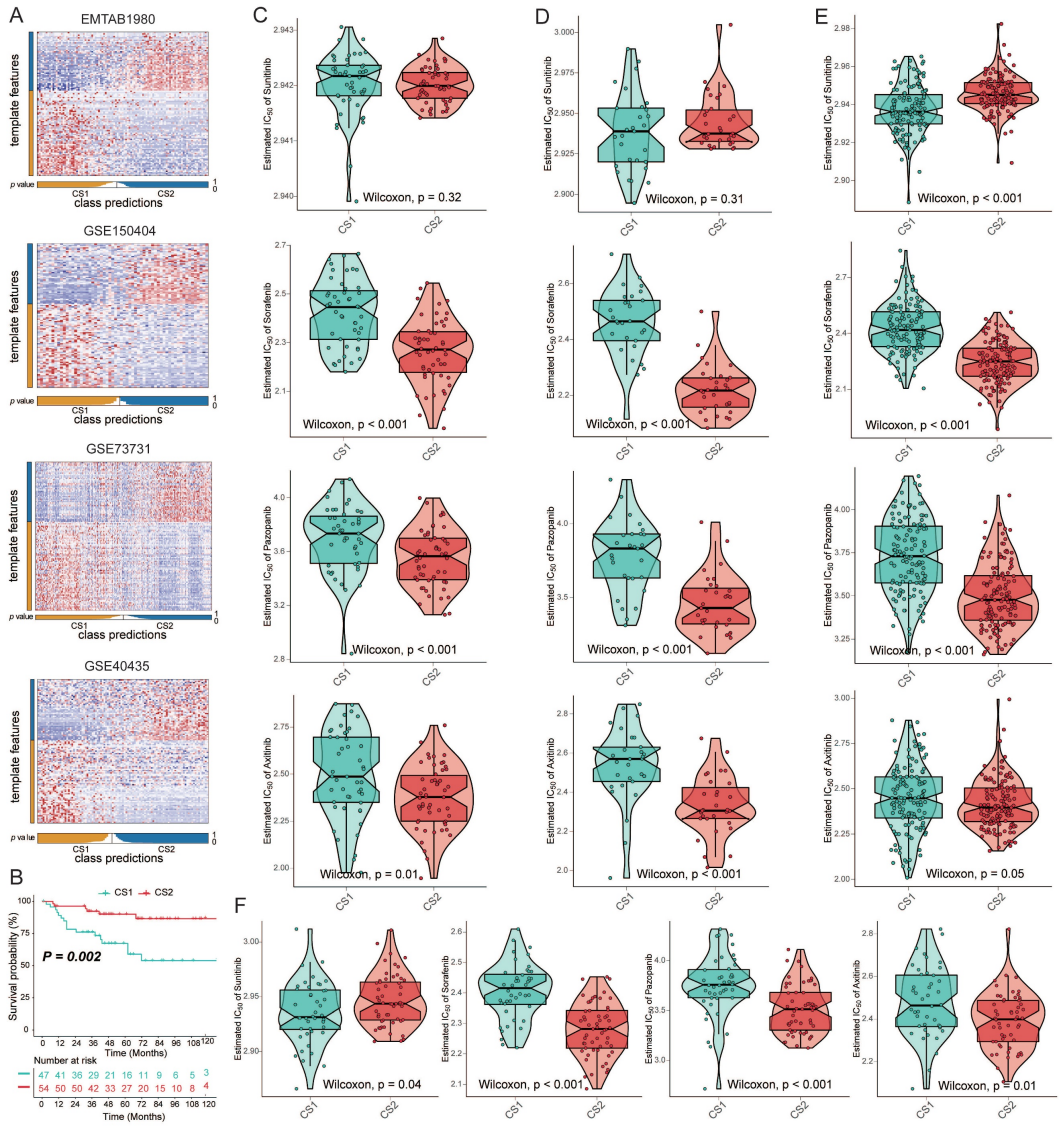
** (A)** Heatmap of NTP in four external cohorts, EMTAB1980, GSE150404, GSE73731 and GSE40435, using subtype-specific upregulated biomarkers identified from the KIRC cohort. **(B)** Kaplan‒Meier survival curve of the two predicted subtypes of the EMTAB1980 cohort. **(C-F)** Box plots of the estimated IC_50_ for sorafenib, sunitinib, axitinib, and pazopanib between the two subtypes.

To verify the reliability of subtype classification, we selected the top 100 specific genes for each subtype that were most specific compared to the other subtypes. We discovered these upregulated biomarkers in CS1 and CS2 by "DESeq2", and adjusted for a significance threshold of P<0.05. No overlap of biomarkers was identified among them. We used four external cohorts, EMTAB1980, GSE150404, GSE73731, and GSE40435, to verify the reliability of the new subtyping. Based on the specific upregulation of biomarkers in subtypes, predictions were made for each cohort using the NTP approach (Fig. 5A). Survival analysis on the EMTAB1980 validation set showed that CS1 had a worse clinical prognosis (Fig. 5B; *P*=0.002). Additionally, we performed a drug sensitivity analysis in the validation cohort. The responses to sorafenib, axitinib, and pazopanib all showed differences between the two subtypes. CS2 showed significantly higher drug sensitivity (Fig. 5C-5F). However, the difference in sensitivity to sunitinib may be questionable, as the difference was not significant in validation data.

## Discussion

KIRC is a genitourinary cancer that mostly originates in the proximal tubular cells of the renal unit 39 and is the most common subtype of RCC. Localized lesions can be cured by partial or radical nephrectomy at the early stage, but one-third of cases still have a risk of relapse or metastasis after surgery. Patients with metastasis have a worse prognosis, with 5- and 10-year survival rates of 5-30% and 0-5%, respectively 40. Clinically advanced or metastatic KIRC remains a tough challenge. Stratifying KIRC based on tumor heterogeneity is very important for finding precision medicine approaches.

In recent years, the accumulation of multi-omics data has provided a valuable resource for subtype analysis. Previous studies have identified KIRC subtypes based on a single omics dataset 11-13, making it difficult to obtain a comprehensive view of tumorigenesis based on its technical limitations. And a single clustering algorithm may not be stable enough for subtype identification. Therefore, the integration of multi-omics information can provide more relevant evidence for biological mechanisms, leading to a deeper understanding of complex biological processes.

In this work, we established stable KIRC subtypes based on ten clustering algorithms. To better comprehend its molecular characteristics, we divided it into two subtypes. Compared with CS2, CS1 has a higher pathological grade and worse prognostic characteristics. Functional and signaling pathway enrichment analysis confirmed that CS1 was enriched in more inflammation-related pathways than CS2, while more oncogenic pathways, such as EMT, TGF-β, Wnt, and other signaling pathways, were also activated in CS1. These signaling pathways were generally considered to be important steps in tumor metastasis and progression and were closely related to poor prognosis 41. In addition, we also identified subtype-related cell subpopulations in single cell data and found that the proportion of malignant cells in CS1-related cell subpopulation was significantly higher.

The molecular mechanisms of KIRC are characterized by genetic diversity and chromosomal complexity. We found more copy number variations in CS1, especially deletions of chromosome 3p, where the von Hippel‒Lindau (VHL) gene is located, which is considered a key genetic event 42. Loss-of-function mutations in the VHL gene induce dysregulation of many VHL-mediated targets, pathways and processes, which is an important step in the development of ccRCC 43. At the same time, mutations in PBRM1 and BAP1, the drivers of tumor evolution, also showed significant differences in our subtypes.

Our study not only stratified patients with KIRC into two subgroups but also provided new insights into predicting tumor sensitivity to targeted therapy and immunotherapy. CS1 may be less responsive to targeted drugs and immunotherapy. We estimated the sensitivity of each sample to targeted therapy based on the IC50 value. The results showed that the CS2 was more sensitive to TKIs. Not all kidney cancer patients respond well to ICIs. Using TIDE predictions, we found CS2 was a more promising subtype with better response to ICIs. Although CS1 showed a higher immune infiltration score, this was likely influenced by suppressive immune features such as Tregs, CD8A+ exhausted T cells and TAMs. The analysis results of bulk and single cell data remained consistent, both indicating that the CS2 subtype had a more active immune infiltration environment, which may inhibit tumor invasion and benefit from immunotherapy.

In summary, no simple and practical method can be used routinely in pathological laboratories to subtype KIRC patients. And it will be promising that we further validate the clinical value of this subtyping prospectively. However, this study also had some limitations. First, the TCGA and single-cell data used in our study mainly came from patients in developed countries, and there is a lack of data from developing countries. Second, although both bulk data and single cell data are from patients in European and American countries, they are not from the same corresponding patients, which may cause our results to be slightly biased.

## Conclusions

Our research is innovative in evaluating classifications of KIRC with multi-omics data as well as multi-clustering approach. We performed a comprehensive molecular characterization on KIRC and integrated single cell data to deeply analyze the heterogeneity at the cellular level. The molecular differences between the observed subtypes may be helpful to provide new biomarkers for particular therapies and open up new possibilities for precision medicine of KIRC patients. Meanwhile, our work of drug sensitivity analysis and immune prediction also revealed potential therapeutic strategies for KIRC precision therapy.

## Supplementary Material

Supplementary figures and tables.

## Figures and Tables

**Table 1:** Baseline characteristics of KIRC participants in the CS1 and CS2

		CS1 (N=113)	CS2 (N=139)	P value
Age (median [IQR])		62.00 [54.00, 70.00]	61.00 [53.00, 72.00]	0.934
Gender (%)	FEMALE	25 (22.1)	59 (42.4)	0.001
	MALE	88 (77.9)	80 (57.6)	
Race (%)	WHITE	105 (92.9)	133 (95.7)	0.229
	Others	7 (6.2)	4 (2.9)	
	Unknown	1 (0.9)	2 (1.4)	
pStage (%)	Stage I	39 (34.5)	76 (54.7)	0.006
	Stage II	10 (8.8)	15 (10.8)	
	Stage III	38 (33.6)	27 (19.4)	
	Stage IV	25 (22.1)	21 (15.1)	
	Unknown	1 (0.9)	0 (0.0)	
Grade (%)	G1	0 (0.0)	4 (2.9)	<0.001
	G2	39 (34.5)	65 (46.8)	
	G3	41 (36.3)	60 (43.2)	
	G4	32 (28.3)	10 (7.2)	
	GX	1 (0.9)	0 (0.0)	
