# Supplementary material for: Integrative analysis of single-cell and bulk multi-omics data to reveal subtype-specific characteristics and therapeutic strategies in clear cell renal cell carcinoma patients
Source: J Cancer. 2024 Oct 18;15(19):6420–33. doi: 10.7150/jca.101451 (PMC11540511; doi:10.7150/jca.101451)
Supplement: Supplementary file 1 — Supplementary figures and tables. [file jcav15p6420s1.zip › Supplementary Materials.pdf]

Supplementary Materials:

Integrative analysis of single-cell and bulk multi-omics data to reveal subtype-specific characteristics and therapeutic strategies in clear cell renal cell carcinoma patients

Xinjia Ruan[1†], Chong Lai[2†], Leqi Li[1†], Xiaofan Lu[1], Dandan Zhang[3], Jingya Fang[1*], Maode Lai[3*], Fangrong Yan[1*]

[1] State Key Laboratory of Natural Medicines, Research Center of Biostatistics and Computational Pharmacy, China Pharmaceutical University, Nanjing 211198, P.R. China.

[2] Department of Urology, The First Affiliated Hospital, Zhejiang University School of Medicine, Hangzhou 310012, P.R. China.

[3] Department of Pathology, Zhejiang University School of Medicine, Hangzhou 310058, P.R. China

[†] These authors have contributed equally to this work.

*** Correspondence:**

Fangrong Yan, Research Center of Biostatistics and Computational Pharmacy, China Pharmaceutical University, Nanjing, PR China, 211198. E-mail: f.r.yan@163.com
Maode Lai, Department of pathology, Zhejiang University School of medicine, Hangzhou 310058, P.R. China. Email: lmd@zju.edu.cn
Jingya Fang, Research Center of Biostatistics and Computational Pharmacy, China Pharmaceutical University, Nanjing, PR China, 211198. E-mail: jyfang1904@163.com

Supplementary Figures

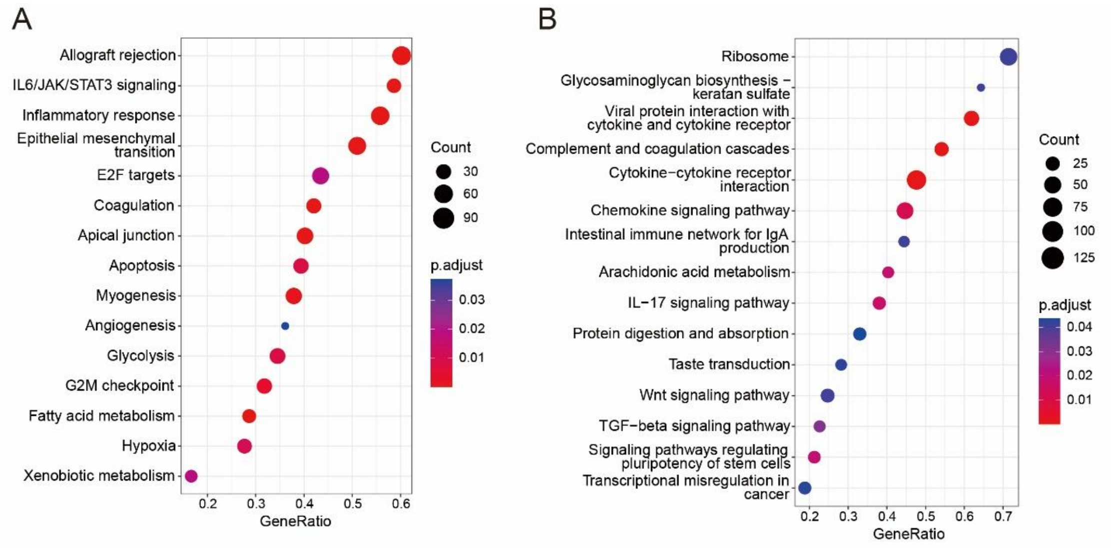

Supplementary Fig. 1. (A) Dot plot for GSEA pathway analysis of the hallmark gene set. The adjusted *P* value and gene count are depicted as dot color and size. **(B)** Dot plot for KEGG pathway analysis and each point in the plot represents a pathway from KEGG analysis.

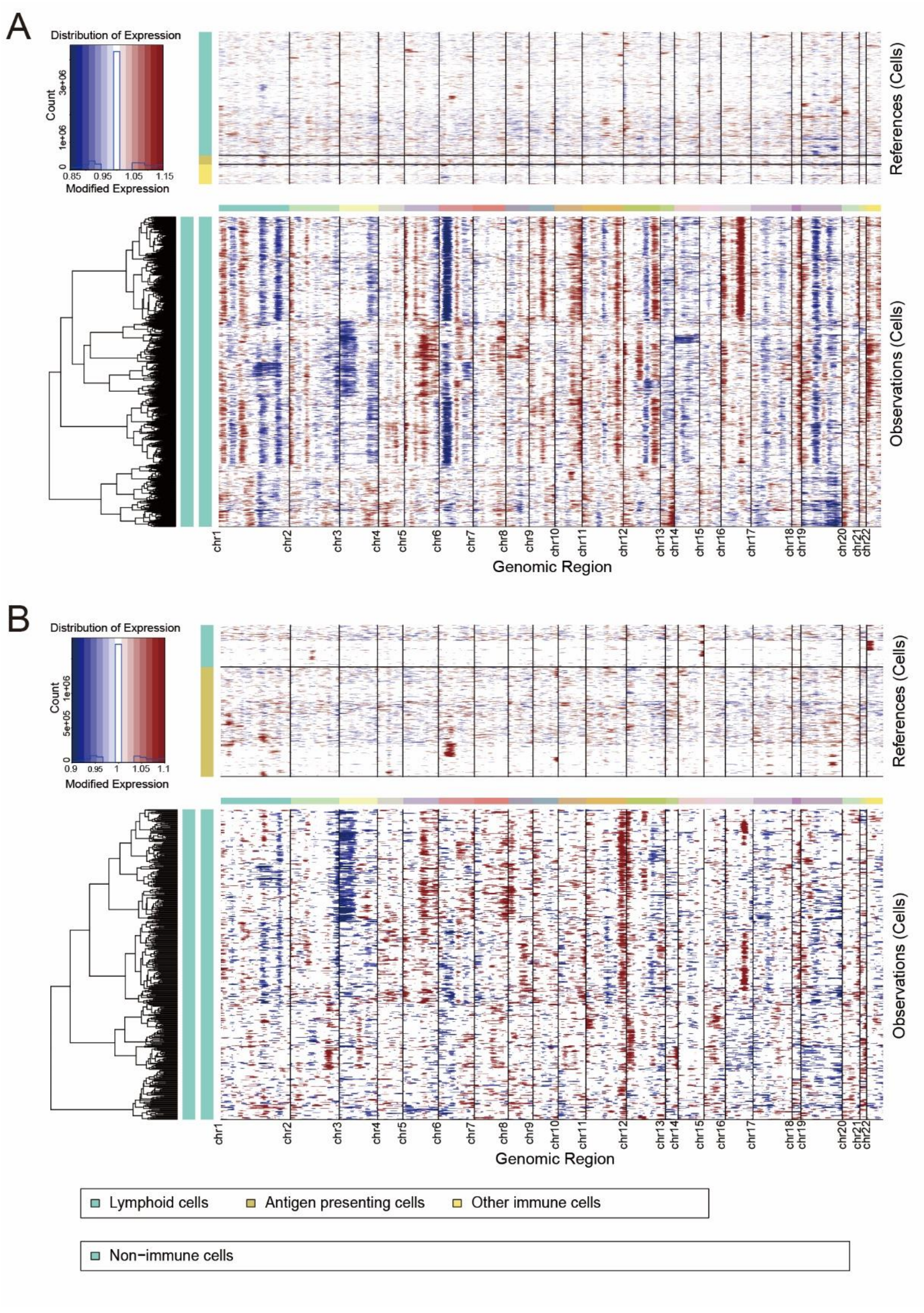

Supplementary Fig. 2. (A) Heatmap showed CNVs of non-immune cells in Scissor_CS1. **(B)** Heatmap showed CNVs of non-immune cells in Scissor_CS2.

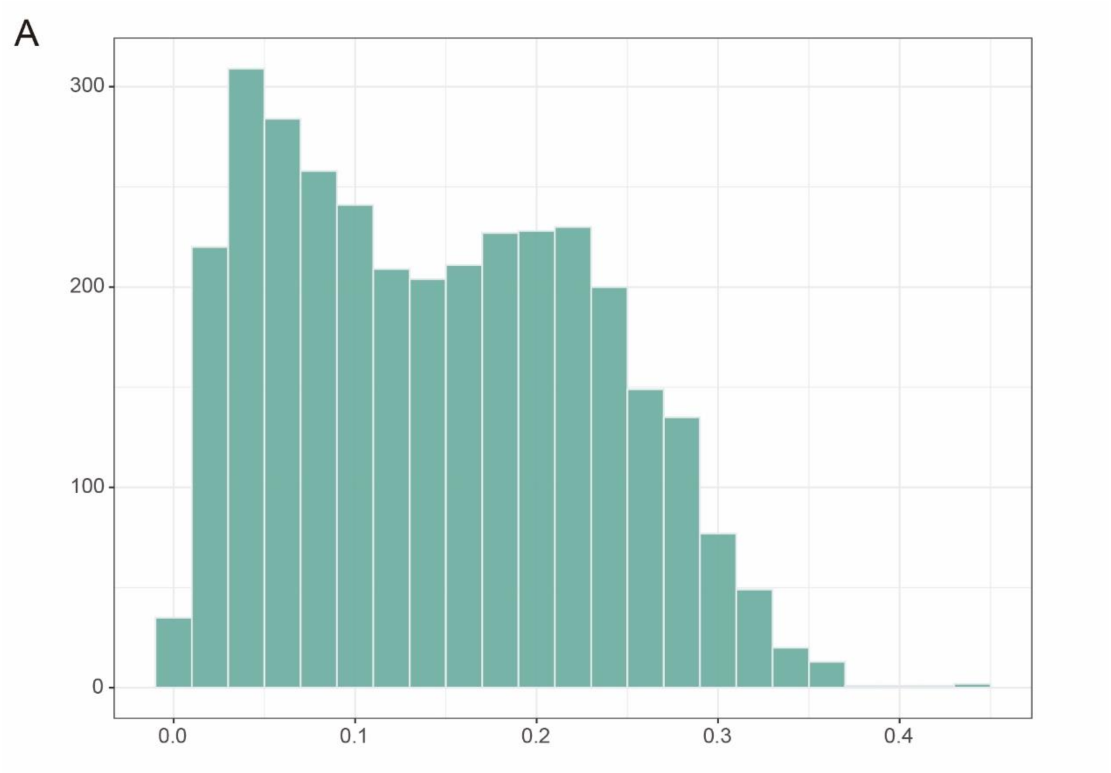

Supplementary Fig. 3. (A) Histogram plot of copy number variation score distribution in non-immune cells.

Supplementary Tables

Supplementary Table S1. Basic information of five external validation cohorts.

Supplementary Table S2. Significantly differentially expressed genes of CS1 and CS2 generated by DESeq2.
